# Effect of Visual Feedback on the Occipital-Parietal-Motor Network in Parkinson’s Disease with Freezing of Gait

**DOI:** 10.3389/fneur.2013.00209

**Published:** 2014-01-09

**Authors:** Priya D. Velu, Tim Mullen, Eunho Noh, Matthew C. Valdivia, Howard Poizner, Yoram Baram, Virginia R. de Sa

**Affiliations:** ^1^Department of Neurosciences, University of California, San Diego, CA, USA; ^2^Cognitive Science Department, University of California, San Diego, CA, USA; ^3^Swartz Center for Computational Neuroscience, University of California, San Diego, CA, USA; ^4^Institute for Neural Computation, University of California, San Diego, CA, USA; ^5^Electrical Engineering Department, University of California, San Diego, CA, USA; ^6^Computer Science Department, Technion, Haifa, Israel

**Keywords:** FOG, visual feedback, occipital lobe, parkinsonian disorders, EEG-fMRI

## Abstract

Freezing of gait (FOG) is an elusive phenomenon that debilitates a large number of Parkinson’s disease (PD) patients regardless of stage of disease, medication status, or deep brain stimulation implantation. Sensory feedback cues, especially visual feedback cues, have been shown to alleviate FOG episodes or even prevent episodes from occurring. Here, we examine cortical information flow between occipital, parietal, and motor areas during the pre-movement stage of gait in a PD-with-FOG patient that had a strong positive behavioral response to visual cues, one PD-with-FOG patient without any behavioral response to visual cues, and age-matched healthy controls, before and after training with visual feedback. Results for this case study show differences in cortical information flow between the responding PD-with-FOG patient and the other two subject types, notably, an increased information flow in the beta range. Tentatively suggesting the formation of an alternative cortical sensory-motor pathway during training with visual feedback, these results are proposed as subject for further verification employing larger cohorts of patients.

## Introduction

Freezing of gait (FOG) is a debilitating phenomenon in a subset of patients with Parkinson’s disease (PD). FOG occurs in 53% of PD patients who are in advanced stages of disease but can occur even in early stages. The freezing episodes usually last a few seconds to a minute, though longer durations are not uncommon (1–4). Behavioral studies have shown that cadence of gait increases and stride length decreases before a freezing episode (5). The timing and activation of the tibialis anterior (TA) and gastrocnemius muscles of the lower leg involved in the starting and swinging phases of gait are abnormally timed and activated (6).

Earth-stationary visual cues are known to improve gait in PD patients. The feedback control effects of inertially driven virtual reality cues generated by a portable device have been found to improve various gait parameters and reduce or eliminate the eventuality of freezing in some PD patients. Additionally, a residual effect was observed that lasted beyond the period of cue presentation. Similar results were seen in a variety of other neurological disorders [reviewed in Ref. (7)]. Other studies have also shown persistent mitigation of FOG symptoms after visual targets are used (8, 9).

The structural and functional neuroanatomical properties of FOG have been studied by using voxel-based morphometry (VBM), single-photon emission computed tomography (SPECT), and functional magnetic resonance imaging (fMRI). VBM showed gray matter atrophy in the frontal and parietal cortices, specifically in the left cuneus, precuneus, lingual gyrus, and posterior cingulate cortex in PD-with-FOG compared to PD-without-FOG and controls, with clinical severity of FOG correlated significantly with gray matter loss in posterior cortical regions (10). SPECT showed decreased brain perfusion in prefrontal, orbitofrontal, and anterior cingulate regions in PD-with-FOG compared with PD-without-FOG (11). In a VR walking task in PD-with-FOG, fMRI showed decreased blood oxygen level-dependent response in sensorimotor regions and an increased response in frontoparietal cortical regions (12). Another fMRI study that used motor imagery of gait as the task found reduced activity in the superior parietal lobule and the anterior cingulate cortex in both patient groups compared to controls, and a statistical trend toward increased activity in the left supplementary motor cortex and right superior parietal lobe in PD-without-FOG compared to PD-with-FOG with ROI analysis (13). Whole brain analysis in the same study revealed increased task related activity in the posterior mid-mesencephalon of PD-with-FOG compared to PD-without-FOG.

A resting state fMRI study showed decreased functional connectivity within a network consisting of the right middle frontal gyrus and angular gyrus and a network consisting of the right-occipito-temporal gyrus in PD-with-FOG compared to PD-without-FOG (14). These areas are regarded as executive-attention and visual networks, respectively, with the executive-attention network recognized as one of the cognitive resting state networks. Yet, how visual cues change neural responses to overcome or prevent FOG has remained an open question.

By using EEG, we were able to examine time varying directed connectivity as opposed to relative levels of activation of different anatomical regions, which allowed for a dynamic picture of effectors and their targets with millisecond resolution. We hypothesized that visual cues result in an increase in information flow from visual and parietal areas to the motor cortex in the pre-movement time period, and that this effect has residual staying power. We used time-series data from EEG recordings of Controls and PD-with-FOG subjects in a task that required them to turn and walk through a doorway. Analysis was performed on the pre-movement period with the idea that the dysfunction in FOG that occurs in the preparation period before any movement is crucial toward understanding the phenomenon. This also allowed us to reduce any confounding effects on the EEG from movement, muscle activity during gait, or cerebral activity due to motor activity, performance, or sensory feedback. The portable EEG permitted ambulation so that the preparation period reflected planning for actual gait rather than motor imagery as in fMRI studies, since a concern in interpreting motor imagery data is that PD patients are not as capable as their age-matched healthy peers in estimating walking during motor imagery tasks (15).

## Methods

### Subjects

Two subjects with PD-with-FOG, one responding (PDr) and one non-responding (PDnr) to visual feedback, and six age-matched healthy individuals (Control, ages 57–75, 1 female) were analyzed. Both PD patients took medications as scheduled so as to remain in the “ON” state throughout the experiment. All subjects read and signed informed consent forms that were approved by the UCSD Human Research Protections Office. Clinical characteristics of both PD patients are specified in Table 1.

**Table 1:** **Clinical characteristics of PD patients**.

	Age	Sex	Handedness	Duration	UPDRS III	H & Y	FOGQ	Medication	MMSE	BDI
PDr	69	F	L	16	50	3	8	Lev, LevR, Pr, Am, Ras	30	2
PDnr	48	M	R	8	40	3	10	Lev, RopXL, Ras,	30	5

*UPDRS III, United Parkinson’s Disease rating scale, motor section (16). Range 0–108. Higher scores indicate greater impairments. Scores of 50 and 40 reflect moderately severe motor impairment*.

*H&Y Stage, Hoehn and Yahr (1967) staging. Range 0–5. Stage 3 indicates moderately severe parkinsonism*.

*FOGQ, freezing of gait questionnaire (17). Raw score range 0–24. Higher scores indicate greater impairment*.

*MMSE, Folstein mini-mental state examination (18). Range 0–30. Higher scores reflect better cognitive performance. Scores below 24 indicate cognitive impairment; thus, neither patient showed dementia*.

*BDI, Beck depression inventory (19). Range 0–63. Higher scores reflect increasing depression. Scores below 10 indicate no or minimal depression. Thus, neither patient showed depression*.

*Scores on the MMSE and BDI were available for PDnr 5 months prior to testing, and for PDr, 2 years prior to testing*.

*Duration: years since first remembered parkinsonian symptom*.

*Medication codes: LevR, Carbidopa/levodopa sustained release; Lev, Carbidopa/levodopa (regular formulation); Pr, Pramipexole; RopXL, Ropinirole extended release; Ras, Rasagiline; Am, Amantadine*.

### Visual feedback

A cellphone-size belt-mounted box containing inertial sensors and a microprocessor generated an earth-stationary visual cue in the form of checkerboard tiles that were displayed by VR glasses and moved in accordance with the patient’s own motion. A lens, centrally embedded between two non-transparent stereo micro-displays, provided a see-through capability for patient safety.

### Task

**Figure 1:**
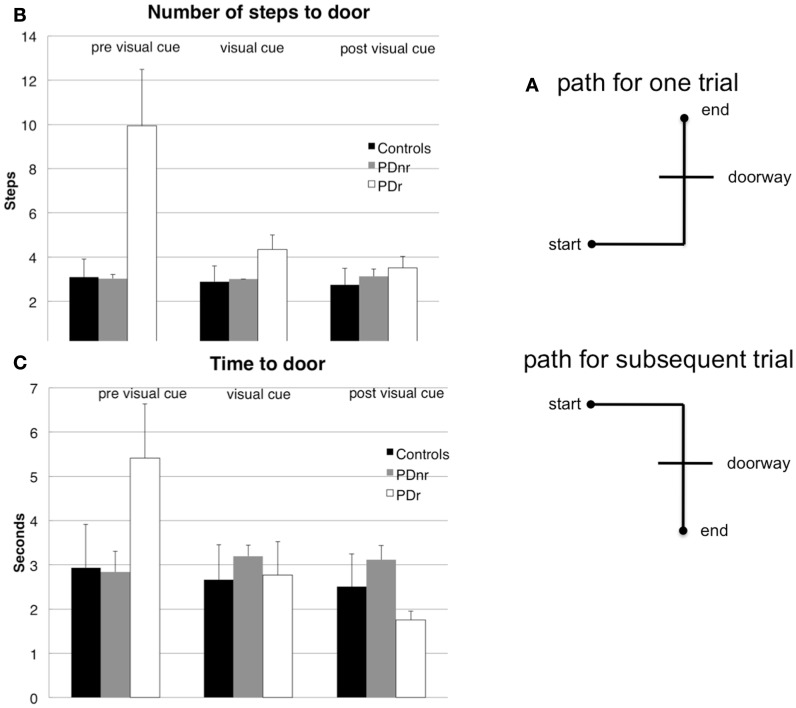
**Subjects had to walk from a starting point, turn, then go through a constructed doorway to finish at the designated endpoint (A)**. The **(B)** number of steps taken to reach the doorway and **(C)** amount of time taken to reach the doorway before cues are shown for before visual cues were presented (pre visual cue or Stage A), during visual cue presentation (visual cue or Stage C), and after visual cues were presented (post visual cue or Stage E). These were plotted for the three subject groups: Control, PDr, and PDnr. For the Control group, standard deviation was calculated from all six controls. For the PDr and PDnr groups, standard deviation was calculated on the individual subject step and time data.

The task consisted of five stages. In stages A, C, and E, the subject walked on a set path that consisted of three maneuvers: (1) start at a designated start spot and walk forward, (2) turn either left or right to approach a doorway, (3) enter and pass through doorway to a designated end spot (Figure 1A). In stages B and D, the subject was seated and asked to remain still with eyes open for 5 min in order to record resting state EEG. Subjects wore the VR glasses throughout the experiment, but visual feedback was only shown in stage C.

A single trial started with an auditory command, “stand still,” to prepare subjects for a “beep” noise that indicated that the subject could begin walking (go cue). The interval between the preparation cue and the go cue was randomly chosen from values of 1250, 1000, or 750 ms. Trials in which subjects started walking before the go cue sounded or that involved any freezing were eliminated from analysis. Each stage lasted until 30 properly performed trials were collected. Subjects could request breaks at any point during a stage in order to sit down, though only one subject (PDr) took advantage of this option.

Subjects wore the EEG cap and electrodes during the entire task. A spotter followed all subjects with a backpack containing a battery pack and EEG amplifier that transmitted data through a 40 feet long fiber optical cable to the recording computer. An additional spotter assisted in experiments with patients for fall prevention.

### Electrophysiological recording

#### Data collection

Continuous EEG and EOG/mastoid/EMG (EXG) were recorded from 64 Ag/AgCl scalp electrodes positioned on a BioSemi nylon head cap according to the 10–20 International System and 8 EXG electrodes placed on the surface of cleaned skin. The signal was amplified with fixed gain BioSemi ActiveTwo amplifiers, band-passed from 0.1 to 100 Hz, and digitized at 512 Hz with 24-bit resolution. The independent software package DataRiver was used to read and record EEG signals as well as to integrate EEG signals with events from the Stim2007 stimulus presentation software. Two EOG electrodes were placed to record eye movements (one on the right outer canthus and one above the right eye). Right and left mastoid electrodes were averaged off-line to serve as reference. To minimize movement artifacts, subjects were encouraged to remain still and look forward until the cue to move was heard. Two electrodes were placed on the anterior tibialis muscle (the first muscle to activate in gait) on each leg to detect premature muscle contraction during trials.

#### Pre-processing and artifact rejection

Pre-processing utilized various functions from the EEGLAB software package (20). Data were referenced to L and R mastoid electrodes and bandpass filtered from 1 to 50 Hz. Data were split into epochs starting 4 s before and ending 4 s after the go cue with −2.75 s to −2.25 s before the go cue used as a baseline. Trials in which leg EMG indicated movement before the go cue sounded and trials that had excessive noise by visual inspection were eliminated. Channels were also visually inspected for noise and removed. These data were then further cleaned using EEGLAB automatic artifact rejection functions that removed channels and epochs that had kurtosis values five standard deviations from the mean kurtosis value. Kurtosis is a fourth moment measure of a probability distribution, and large positive excess kurtosis values indicate an increase in peakedness of the distribution whereas large negative excess kurtosis values indicate an abnormally flat shape in the distribution. In EEG, these may represent undesirable artifacts in the data.

To remove eye and electronic artifacts, Independent Component Analysis (ICA) was performed for each subject on the pre-processed and cleaned data from all three walking stages concatenated to form one dataset. The resulting independent components (ICs) were then analyzed by an automatic algorithm from the ADJUST plug-in for EEGLAB. This algorithm identified ICs for elimination by looking for stereotyped spatial and temporal features present in eye blinks, eye movements, and generic discontinuities such as impedance fluctuations or electronic device interference.

### Behavioral recording

#### Data collection

The amount of time the subject took to reach the doorway from the starting position and that the subject took to pass through the doorway and to stop at the designated end spot were recorded by an observer with a stopwatch. The corresponding number of steps taken by the subject at these timed portions were counted and reported by the spotter holding the backpack.

### Pre-movement EEG analysis using SIFT

EEG analysis was focused on spectral power and connectivity in a network composed of occipital (Oz), parietal (P4), and motor (Cz) channels. These channels were chosen based on anatomical findings from prior fMRI and PET experiments on paradoxical gait in PD (13, 21). Power spectra and connectivity measures were obtained using a multi-trial sliding window adaptive multivariate vector autoregressive (AMVAR) modeling approach applied to the non-stationary channel time series.

#### Pre-processing

For optimal model fitting, data went through several pre-processing steps. They were first down-sampled to 128 Hz from 512 Hz in order to minimize the number of coefficients required to adequately fit a model, as increased model order leads to increased variability of spectral and causal estimates (22). Then, piecewise linear de-trending was applied using a least squares fit to eliminate remaining drift in the data. Finally, data were normalized by point-wise subtraction of the ensemble mean and division by ensemble standard deviation over all trials and the subtraction of the temporal mean and division by the temporal standard deviation for each trial.

#### Model fitting

The Vieira–Morf algorithm performs better than Arfit and Levinson algorithms for small sample sizes (23) and was used for all AMVAR fitting in this paper. The window length was set to 1 s and the step size to 0.01 s. Model order (here, 10) was selected by minimizing Hannan–Quinn criterion, which optimizes a tradeoff between the prediction error of the model and the number of freely estimated parameters in the model (24).

#### Model validation

The AMVAR model was validated using whiteness, consistency, and stability and stationarity measures. Whiteness measures the amount of correlation left in the residuals of the model; an ideal fit has no correlation structure left in the residuals. A portmanteau test for whiteness, specifically the Li–Mcleod which is considered the most conservative, was used to determine if residuals were white (24). Consistency of the model was determined by generating an ensemble of simulated data of equal dimensionality as the original data using the AMVAR model and calculating the auto and cross correlations between all variables to determine if the generated data had at least 85% similar correlation structure to the real data (25). Stability implies stationarity, and stability in an M-dimensional AMVAR model with order p can be checked by ensuring that the eigenvalues of the (Mp × Mp) augmented coefficient matrix have moduli less than 1 (24). This was done by using a stability index based on the log of the largest eigenvalue of the coefficient matrix.

#### Information flow analysis

Connectivity between nodes in networks can be structural, functional, and effective in nature (26). Here we attempted to map effective connections, or causal interactions, between different areas of the brain by calculating directed coherence. There are several causal estimators that can be derived from the AMVAR model coefficients. Here we used renormalized partial directed coherence (rPDC) as it provides a scale-free estimator, avoids arbitrary normalization by inflow or outflow, and provides a constant (frequency-independent) point-wise significance threshold (27).

Nonparametric significance thresholds on between-condition differences in power and rPDC were obtained using an Efron bootstrap approach. In brief, for each *T*-trial dataset, a surrogate dataset was constructed containing *T* trials randomly sampled with replacement from all trials. The surrogate dataset was then subjected to the aforementioned pre-processing and modeling procedure. This procedure was repeated 750 times yielding empirical distributions of power and rPDC for each time window and frequency bin. For each time-frequency “pixel,” a pointwise two-sided empirical *p*-value for rejecting the null hypothesis of equal power or rPDC between any two conditions was then obtained by computing the quantile at which zero occurs in the between-condition distribution of surrogate differences. Finally, pointwise significance estimates were corrected for multiple comparisons across time, frequency, and channel (pair) using the Benjamini-Hochberg False Discovery Rate procedure and thresholded at *p* = 0.05.

## Results

Behavioral measures showed marked decrease in the time and number of steps taken to reach and exit the doorway in stage C in the patient that responded to visual feedback (PDr) compared to the control subjects and the patient that did not respond to visual feedback (PDnr) (Figures 1B,C). PDr retained these behavioral effects in stage E suggesting that there are residual effects from prior feedback.

**Figure 2:**
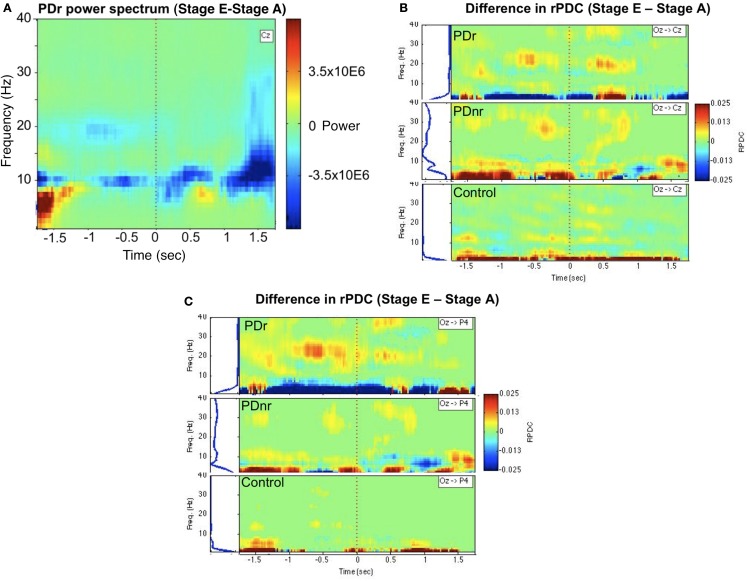
**Between-condition differences in power and connectivity for patients and a representative subject for the Control group (57 M)**. **(A)** Frequency vs. time representation of difference in activity between stages E and A at channel Cz for PDr indicates decreased power in the beta frequency range. This was not seen in PDnr or Control. Blue indicates a decrease in power while red indicates an increase in power. **(B)** Renormalized partial directed coherence (rPDC) is a measure of direction specific information flow from one site to another. Occipital region (Oz) shows increased rPDC to motor region (Cz) in the beta frequency range before and after the cue only in PDr and PDnr, with a higher magnitude and smaller frequency range in PDr. Similar results were seen in the difference in activity between stages C and A, but we show stage E to stage A to avoid any confound from neural responses to the presence of visual cues. **(C)** A similar but more pronounced effect is seen in the rPDC from occipital region to parietal region (P4).

The EEG spectra showed power differences in the delta (0–4 Hz), alpha (8–12 Hz), and beta (12–30 Hz) frequency bands when stages were compared. Of particular interest was the decrease in power in the 18–22 Hz range in PDr after visual cues were given (Figure 2A). There was also increased information flow from Oz to Cz (Figure 2B) and Oz to P4 (Figure 2C) in the beta range in PDr. Delta and alpha band powers increased as the task progressed in PDnr and Control, but decreased for PDr.

## Discussion

Recent studies in PD patients with deep brain stimulation (DBS) have demonstrated the existence of cortico-subthalamic networks that differ in dominant frequency and spatial location (28, 29). One such network exhibits a decrease in dominant beta frequency power between the supplementary motor area (SMA) and the subthalamic nucleus (STN) during voluntary movement compared to rest (29). Synchronization in cortical beta frequency in healthy subjects has been postulated to favor existing motor state over novel movement (30), and in PD patients off therapy vs. on therapy it is excessive, with the degree of synchrony correlated to the level of motor impairment (31).

The decreased power and increased information flow in the beta band in PDr in stage E compared to stage A suggest a correlation between the presentation of visual cues and decrease in beta band oscillations to allow for movement to occur.

Increases in delta and alpha band power in PDnr and Control may have resulted from boredom or inattention as the task progressed (32). Conversely in PDr, the spectral changes in these bands may be a result of greater motivation and reward in the task as the subject improved in gait parameters.

One caveat is that the alleviation of FOG with visual cues does not imply that the FOG has a perceptuovisual origin. Visual cues may simply provide an alternate cortico-cerebellar pathway that can compensate for the motor impairment of FOG regardless of its true origination. Recently a group demonstrated the feasibility of reproducing FOG in MPTP-treated macaque monkeys, suggesting that lesion studies may 1 day define the anatomical and pathophysiological correlates of FOG.

The results of the present case study, suggesting that visual feedback cues affect activity and information flow in nodes of an occipital-parietal-motor network, provide possible insights into cortical neural processes underlying gait improvement with visual feedback in FOG.

Given the limited number of participants, these results should be regarded as tentative observations, to be further validated in larger cohorts of patients. Furthermore, as connectivity analysis was performed on the sensor level, we cannot rule out the possibility that volume conduction contributed to the causal effects we observed. Source-level level analysis is a future step that we intend to take. The novelty of these observations and their potential implications on intervention by visual feedback encourage the authors to continue these studies and to suggest further examination and possible extension of these intriguing results by others.

## Conflict of Interest Statement

Yoram Baram is the developer of the visual feedback device used in this study. The other co-authors declare that the research was conducted in the absence of any commercial or financial relationships that could be construed as a potential conflict of interest.
